# Electrophysiological evidence for change detection in speech sound patterns by anesthetized rats

**DOI:** 10.3389/fnins.2014.00374

**Published:** 2014-11-17

**Authors:** Piia Astikainen, Tanel Mällo, Timo Ruusuvirta, Risto Näätänen

**Affiliations:** ^1^Department of Psychology, University of JyväskyläJyväskylä, Finland; ^2^Centre for Learning Research, University of TurkuTurku, Finland; ^3^Department of Teacher education/Rauma Unit, University of TurkuRauma, Finland; ^4^Institute of Psychology, University of TartuTartu, Estonia; ^5^Center of Functionally Integrative Neuroscience, University of ÅrhusÅrhus, Denmark; ^6^Cognitive Brain Research Unit, Institute of Behavioral Sciences, University of HelsinkiHelsinki, Finland

**Keywords:** local-field potentials, pattern perception, auditory cortex, rat, mismatch negativity, speech

## Abstract

Human infants are able to detect changes in grammatical rules in a speech sound stream. Here, we tested whether rats have a comparable ability by using an electrophysiological measure that has been shown to reflect higher order auditory cognition even before it becomes manifested in behavioral level. Urethane-anesthetized rats were presented with a stream of sequences consisting of three pseudowords carried out at a fast pace. Frequently presented “standard” sequences had 16 variants which all had the same structure. They were occasionally replaced by acoustically novel “deviant” sequences of two different types: structurally consistent and inconsistent sequences. Two stimulus conditions were presented for separate animal groups. In one stimulus condition, the standard and the pattern-obeying deviant sequences had an AAB structure, while the pattern-violating deviant sequences had an ABB structure. In the other stimulus condition, these assignments were reversed. During the stimulus presentation, local-field potentials were recorded from the dura, above the auditory cortex. Two temporally separate differential brain responses to the deviant sequences reflected the detection of the deviant speech sound sequences. The first response was elicited by both types of deviant sequences and reflected most probably their acoustical novelty. The second response was elicited specifically by the structurally inconsistent deviant sequences (pattern-violating deviant sequences), suggesting that rats were able to detect changes in the pattern of three-syllabic speech sound sequence (i.e., location of the reduplication of an element in the sequence). Since all the deviant sound sequences were constructed of novel items, our findings indicate that, similarly to the human brain, the rat brain has the ability to automatically generalize extracted structural information to new items.

## Introduction

The ability to detect abstract grammatical rules, i.e., principles that govern speech sound streams, is essential for learning a language. To investigate the infants' ability to extract abstract algebraic rules, Marcus et al. (1999) familiarized infants to sequences of syllables (or sentences) that followed a particular “grammatical” rule (e.g., “ga ti ga” for ABA). During the test, infants were observed to be more attentive to sequences that were grammatically inconsistent (e.g., “wo fe fe,” which is ABB) than to those sequences that were consistent with grammatical rules (e.g., “wo fe wo”). Because the test sentences were different to those used in the training phase, the authors concluded that infants can extract an abstract rule and generalize it to novel instances. Also, detection of ABB and AAB structures were compared, and it was found that even if both structures have a reduplication element, the infants paid more attention to the inconsistent patterns.

It is not known, however, whether the ability to extract grammatical rules from speech sounds only applies to human linguistic cognition or whether this cognitive element has originally evolved for other, more general purposes. In the latter case, these skills could also be found in non-human animal species.

It is known that non-human animal species can process speech up to a certain level of cognitive complexity. Speech sound discrimination has been demonstrated in various animal species neurophysiologically (e.g., Dooling and Brown, 1990 in birds; Kraus et al., 1994 in guinea pigs, Ahmed et al., 2011 in rats), and on a behavioral level (e.g., Engineer et al., 2008 in rats; Sinnott et al., 1976 in monkeys; Sinnott and Mosteller, 2001 in gerbils). Also, word segmentation based on transitional probabilities has been demonstrated, on a behavioral level, in rats (Toro and Trobalon, 2005) as well as in cotton-top tamarins (Hauser et al., 2001). Extraction of grammatical rules (i.e., structural patterns) from speech sounds in non-human species has been studied in tamarin-monkeys and rats with similar stimulus conditions as applied originally by Marcus et al. (1999). The report concerning tamarin-monkeys (Hauser et al., 2002) was later retracted (Retraction notice, 2010). In rats, no evidence of pattern extraction was found (Toro and Trobalon, 2005).

It might be, however, too early to conclude that rats are not able to extract structural patterns from three-syllabic speech sequences, as were applied in a classic study by Marcus et al. (1999) in infants. Since there is evidence in rats of representing abstract rules from pure tones (Murphy et al., 2008), this issue should be further explored. To the present study we applied a neurophysiological mismatch response (MMR), a measure of automatic cognition, which is the equivalent of the human electrophysiological response called mismatch negativity (MMN; Näätänen et al., 1978, 1997, 2010). MMR can reflect auditory cognition before its behavioral manifestation (e.g., Tremblay et al., 1998). Based on this method, we have previously demonstrated that the rat's brain is able to detect changes in abstract auditory features, such as melodic patterns in tone-pairs (Ruusuvirta et al., 2007) and in combinatory rules between frequency and intensity of the sound objects (Astikainen et al., 2006, 2014). Rats also make representations of spectro-temporally complex sounds such as speech sounds in their brains, and they can detect changes in these sounds based on the content of the transient memory (Ahmed et al., 2011). Rats, anesthetized with urethane have been used in these studies as urethane is known to largely preserve the awake-like function of the brain (Maggi and Meli, 1986).

In the present study, capitalizing on the above mentioned studies, we recorded local-field potentials (LFPs) from the dura, above the auditory cortex in urethane-anesthetized rats. We presented the animals with a series of synthesized speech sounds. The stimulus series (modified from Marcus et al., 1999) consisted of several different sequences consisting of three pseudowords (called sentences here). Ninety percent of the sentences followed a specific pattern structure (“standards”). Acoustically novel sentences were introduced (“deviants”) rarely (10% of the sentences) and randomly in the sequences. Deviant sentences were of two different types: 1) “pattern-obeying deviants” that shared the pattern structure of the standard sentences but deviated from them physically, and 2) “pattern-violating deviants” that differed from the standards physically but also presented a different pattern structure. We expected to observe an early MMR to be triggered by the first pseudoword for both types of deviant sentences due to their acoustical differences from the standard pseudowords. We also expected to observe a later MMR to be triggered by the second word in the pattern-violating deviant sentences. This would indicate that the syntax-like rule, carried by the standard patterns, was extracted by the animals' brains.

## Materials and methods

### Subjects

The subjects were 14 male Sprague-Dawley rats from Harlan Laboratories (England, UK), weighing 410–500 g and aged between 13 and 18 weeks at the time of the individual recordings. The animals were housed in standard plastic cages, in groups of 2–4, under a controlled temperature and subjected to a 12 h light/dark cycle, with free access to water and food pellets in the Experimental Animal Unit of the University of Jyväskylä, Jyväskylä, Finland. The experiments were approved by the Finnish National Animal Experiment Board, and carried out in accordance with the European Communities Council Directive (86/609/EEC) regarding the care and use of animals used for experimental procedures. The license for the present experiments has been approved by County Administrative Board of Southern Finland (Permit code: ESLH-2007-00662).

### Surgery

All surgical procedures were done under urethane (Sigma Chemicals, St Louis, MO, USA) induced anesthesia (1.2 g/kg dose, 0.24 g/ml concentration, injected intraperitoneally). Supplemental doses were injected if the required level of anesthesia was not obtained. The level of anesthesia was monitored by testing the withdrawal reflexes. The anesthetized animal was moved into a Faraday cage and mounted in a standard stereotactic frame (David Kopf Instruments, Model 962, Tujunga, CA, USA). The animal's head was fixed to the stereotaxic frame using blunt ear bars. Under additional local anesthesia (lidocaine 20%, Orion Pharma, Espoo, Finland), the skin was removed from the top of the head and the skull revealed. Positioned contralaterally to the recording site, two stainless steel skull screws (0.9 mm diameter, World Precision Instruments, Berlin, Germany) fixed above the cerebellum (AP −11.0, ML 3.0) and frontal cortex (AP +4.0, ML 3.0) served as reference and ground electrodes, respectively. A headstage, composed of a screw and dental acrylic, was attached to the right prefrontal part of the skull to hold the head in place and allow removal of the right ear bar. A unilateral craniotomy was performed in order to expose a 2 × 2 mm region over the left auditory cortex (4.5–6.5 mm posterior to the bregma and 2–4 mm lateral to the bony ridge between the dorsal and lateral skull surfaces) for the placement of the recording electrode. The level of anesthesia was periodically monitored throughout the whole experiment. Animals were rehydrated with a 2 ml injection of saline under the skin every 2 h. After the surgery, the right ear bar was removed and recording started. After the experiment, the animals were further anesthetized with urethane and then put down by cervical dislocation.

### Recording

Local-field potentials in response to auditory stimuli were recorded with a teflon-coated stainless steel wire (200 μm in diameter, A-M Systems, Chantilly, VA) positioned on the dura surface above the left auditory cortex. Continuous electrocorticogram was primarily amplified 10-fold, by using the AI 405 amplifier (Molecular Devices Corporation, Union City, CA, USA), high-pass filtered at 0.1 Hz, 200-fold amplified, and low-pass filtered at 400 Hz (CyberAmp 380, Molecular Devices Corporation), and finally sampled with 16-bit precision at 2 kHz (DigiData 1320A, Molecular Devices Corporation). The data were stored on a computer hard disk using Axoscope 9.0 data acquisition software (Molecular Devices Corporation) for later off-line analysis.

### Stimuli

Synthesized human male voice speech sounds which consisted of five formants, were created using Mikropuhe 5-software (Timehouse, Helsinki, Finland). The speech sound stream consisted of consonant-vowel syllables (words) that were 100 ms in duration. These were presented in groups of three (modified from Marcus et al., 1999). There was a 50-ms pause between each consecutive word, within the sentences, and 100-ms pause between the sentences.

One of the two stimulus blocks (1 or 2) was presented in each animal (*n* = 7 for both blocks, see Table 1). In each block, 90% of the sentences (“standards”) followed a specific structure. For one block, this structure was of AAB type (two identical words followed by a different word) and for the other block of ABB type (one word followed by two identical words). In each block, one structure was assigned to the standards (16 different variants, *p* = 0.9) and the other structure for the deviants (*p* = 0.1).The deviants were of two different types: (1) “pattern-obeying deviants” (2 variants, *p* = 0.05) that physically differed from the standards but obeyed the structure of standard sentences and (2) “pattern-violating deviants” (2 variants, *p* = 0.05) that differed from the standard sentences, both physically and in respect of the pattern. Since all the stimulus types included a repetition of an element, they were not possible to differentiate by detecting only this property of the stimulus. The sentences were ordered in a pseudorandom fashion with the restriction that consecutive deviants were separated by at least two standards. There were a total of 996 stimulus sequences in one stimulus block.

**Table 1:** **Stimulus categories and sequence variants**.

	**Stimulus categories**	**Sequence variants**
Stimulus block 1	Standard “A-A-B” (90%)	LE-LE-JE; LE-LE-WE; LE-LE-DI; LE-LE-LI; WI-WI-JE; WI-WI-WE; WI-WI-DI; WI-WI-LI; JI-JI-JE; JI-JI-WE; JI-JI-DI; JI-JI-LI; DE-DE-JE; DE-DE-WE; DE-DE-DI; DE-DE-LI
	Pattern-obeying deviant “A-A-B” (5%)	BA-BA-BO, KO-KO-GE
	Pattern-violating deviant “A-B-B” (5%)	BA-PO-PO, KO-GA-GA
Stimulus block 2	Standard “A-B-B” (90%)	LE-JE-JE; LE-WE-WE; LE-DI-DI; LE-LI-LI; WI-JE-JE; WI-WE-WE; WI-DI-DI; WI-LI-LI; JI-JE-JE; JI-WE-WE; JI-DI-DI; JI-LI-LI; DE-JE-JE; DE-WE-WE; DE-DI-DI; DE-LI-LI
	Pattern-obeying deviant “A-B-B” (5%)	BA-BO-BO, KO-GE-GE
	Pattern-violating deviant “A-A-B” (5%)	BA-BA-PO, KO-KO-GA

One of the structures (AAB or ABB, in different stimulus blocks) was assigned to standards and pattern-obeying deviants. The other structure was assigned to pattern-violating deviants. Sixteen variants of standard sentences were used in both stimulus blocks to exclude the possibility of standards being memorized by the brain as individual objects. In both type of deviants, two variants were applied per stimulus block. The percentages refer to the proportion of each of the stimulus categories out of the total number of sentences (996).The stimulus block 1 was applied for one animal group (n = 7) and stimulus block 2 for the other animal group (n = 7).

The speech sounds were played from a PC via an active loudspeaker system (Studiopro 3, M-audio, Irwindale, CA, USA). The stimulation was presented with the loudspeaker system directed toward the right ear of the animal at a distance of 20 cm. In all conditions, the sound pressure level for each tone was 70 dB, as measured with a sound level meter (type 2235, Bruel and Kjaer, Nærum Denmark) with C-weighting (optimized for 40–100 dB measurement) in the vicinity of the animal's right pinna during the recording.

### Analysis

The data were off-line filtered at 0.1–30 Hz (24 dB/octave roll off). Data of the two animal groups (stimulus blocks 1 and 2) were averaged. Sweeps from 50 ms before to 500 ms after each stimulus onset were segmented. In order to have same amount of standard and deviant responses in the analysis, only the responses to the standard sentences immediately preceding the deviant sentences were analyzed. The averaged waveforms were then baseline-corrected. The baseline correction was calculated for the period of -50 to 0 ms relative to the second word in the sentence since the change in the pattern occurred at that time in the pattern-violating deviants.

First, the timing of the MMR was investigated by applying point-by-point 2-tailed paired *t*-tests to compare local-field potential amplitudes for the standard and deviant sentences. *P*-values smaller than or equal to 0.05 for at least 20 consecutive sample points (i.e., for the period of 10 ms) were required for the difference in local-field potentials to be considered robust. Next, ANOVA with factors stimulus type (standard vs. deviant) and deviant type (pattern-obeying deviant vs. pattern-violating deviant) for the MMR specific to the pattern-violating deviant sentences was applied. For the ANOVA, mean amplitude values were extracted from the latency range of the significant differential response indicated by the point-by-point *t*-tests. Partial eta squared values present effect size estimates for ANOVA and Cohen's *d* for *t*-tests.

## Results

**Figure 1:**
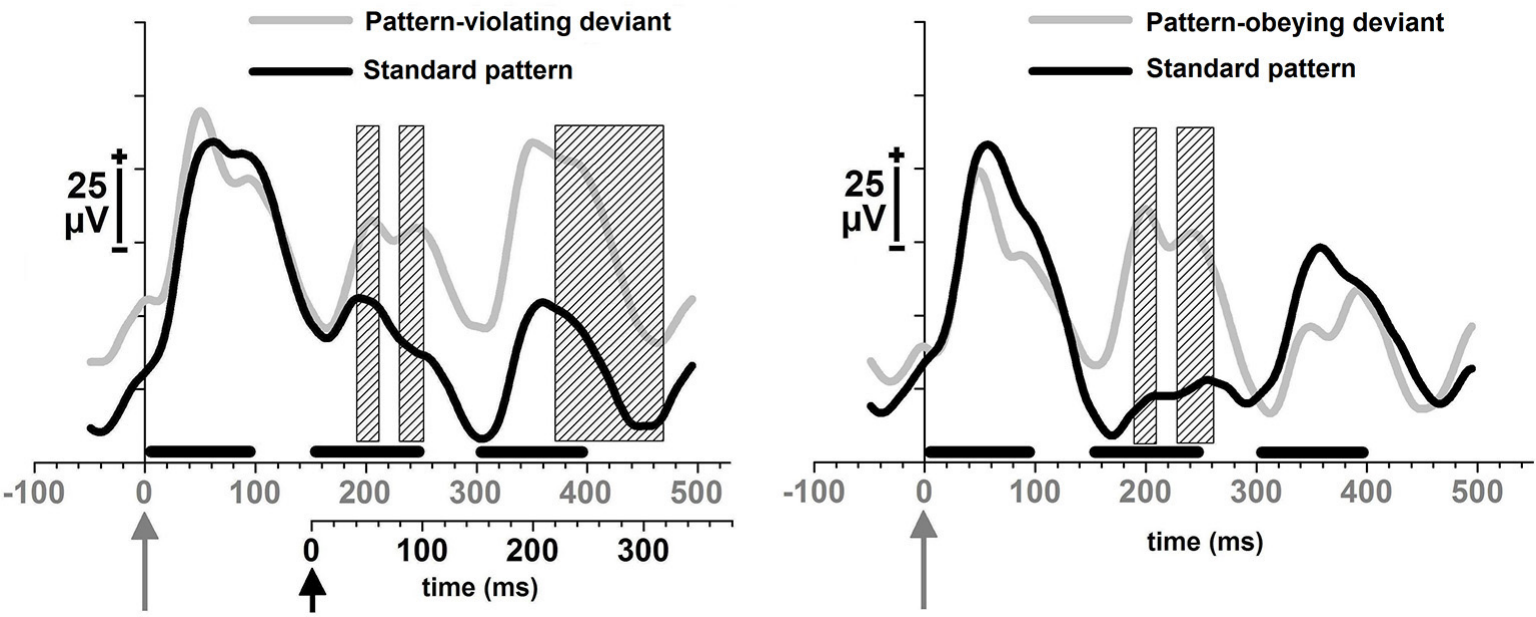
**Local-field potentials in response to pseudo-sentences**. Responses to pattern-violating deviants and standard sentences immediately preceding them (left); and responses to pattern-obeying deviants and standard sentences immediately preceding them (right). The horizontal black bars represent each of the three pseudowords of 100 ms in duration. The triplets were presented at 150 ms stimulus-onset-asynchrony. The gray arrow in figures refers to the onset of the first word of a deviant sentence that physically differed from the standards; and the black arrow in the figure on the left refers to the onset of the structural change present only in the pattern-violating deviants. The two different time scales at the bottom of the left figure refer to the two different onsets of the different deviances in the pattern-violating deviant sentences (onset of the physical difference—the gray time line; onset of the pattern-related difference—the black time line). Shaded rectangles illustrate the time windows of significant amplitude differences (*p* < 0.05) between the two waveforms as indicated by point-by-point *t*-tests.

The first MMR, i.e., an amplitude difference in local-field potentials, between the standard and the deviant sentences, was found for both the pattern-violating deviant sentences (Figure 1, left) and the pattern-obeying deviant sentences (Figure 1, right). This first MMR for the pattern-violating deviant sentences, was significant at 194–213 ms after the sentence onset, [*t*_(13)_ = 2.2–2.7, *p* = 0.020–0.047], and at 231.5–251 ms after the sentence onset, [*t*_(13)_ = 2.2–2.3, *p* = 0.039–0.050]. For the pattern-obeying deviant sentences the corresponding latency ranges were 187.5–206.5 ms after the sentence onset, [*t*_(13)_ = 2.155–2.379, *p* = 0.033–0.050], and 228–261 ms after the sentence onset, [*t*_(13)_ = 2.2–2.8, *p* = 0.016–0.048].

The second MMR was found only for the pattern-violating deviant sentences, in which the second word at a low probability (probability 0.05) violated the pattern that the rest of the sentences followed (probability 0.95). The latency for this MMR second was 217.5–316.5 ms from the onset of the second word, [*t*_(13)_ = 2.2–3.6, *p* = 0.003–0.050] (Figure 1, left).

**Figure 2:**
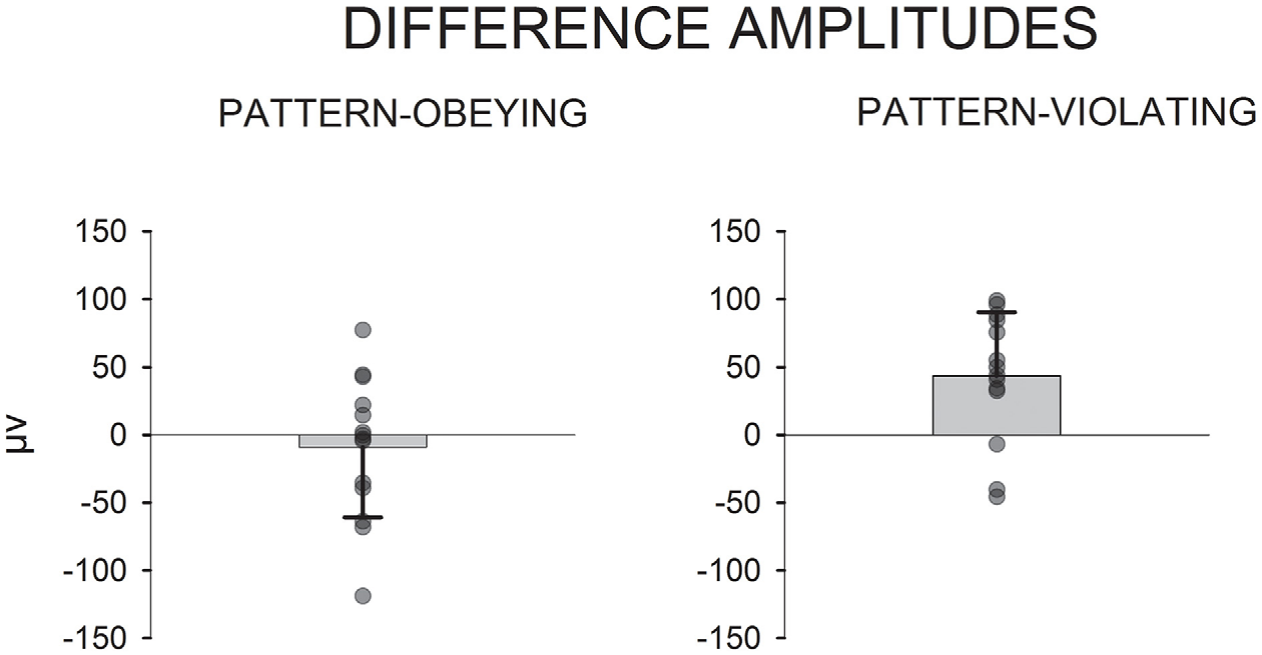
**Mean amplitude values, standard deviation and scatterplots for the individual animals' amplitude values for the second MMR (217.5–316.5 ms from the onset of the second word)**. Differential LFPs (deviant - standard) to pattern-obeying and pattern-violating deviant sentences.

Next, an ANOVA comparing the responses to the pattern-violating and pattern-obeying deviants and their consecutive standards in the time window in which the second MMR was found (i.e., 217.5–316.5 ms from the onset of the second word) was conducted. Significant interaction effect of stimulus type × deviant type was found, [*F*_(1, 13)_ = 8.7, *p* = 0.011, η^2^_p_ = 0.401]. Main effects were non-significant. Responses to pattern-violating deviant sequences and those to the preceding standard sequences differed significantly, [*t*_(13)_ = 3.5, *p* = 0.004, *d* = 1.02]. The corresponding difference was non-significant for the pattern-obeying deviants and preceding standards, [*t*_(13)_ = 0.7, *p* = 0.525, *d* = 0.23]. Figure 2 depicts the mean amplitude values, standard deviation, and individual subjects' amplitude values for the differential responses.

## Discussion

Both types of deviant sentences, pattern-obeying and pattern-violating deviants, were detected from among the repeated standard sentences in the rat brain as indexed by the electrophysiological mismatch response. The earlier difference starting at 187.5 ms, after the sentence onset, was most probably elicited by the physical novelty of the deviant sounds; since the probability for the each standard variant was 22.5% and that of the deviant variants was 5%. An additional mismatch response, starting at 217.5 ms from the onset of the pattern change, was specifically found for the deviant sound sequences that were different in pattern structure from the frequently presented standard sequences. This finding suggests that anesthetized rats are able to extract structural patterns from speech stream that is carried out at a fast pace, and generalize this information to new items (since the deviant sentences differed physically from the standard sentences). Namely, in order to detect the pattern-violating deviant sequences, the brains of the animals needed to make a representation of the structure in the frequently presented “standard” sequences (Näätänen et al., 2001, 2010).

There is previous evidence of non-human animals' ability to extract grammatical rules from speech sounds. Common marmosets (New World monkeys) detected the grammatical differences based on simpler learning strategies than Rhesus monkeys (Old Wold monkeys) (Wilson et al., 2013). Similar ability for rule extraction has been previously reported from sinusoidal sounds in rats (Murphy et al., 2008) and from speech-specific calls in song birds (e.g., Gentner et al., 2006). In human infants, there is evidence that they learn more easily rule-like regularities from speech than from other auditory material (Marcus et al., 2007). It is not known whether this preference is related to linguistic potential in an infant's brain, familiarity of the speech sounds, or some other factors. Future studies in non-human animals could enlighten this issue.

In the present study we tested the rats' ability to detect pattern violation is speech sound sequences that all included a repetition of an element. Therefore, they were not possible to differentiate by detecting only this property of the stimulus. On the other hand, the generalization of the present results may be restricted to stimuli in which the pattern is defined as a repetition of an element and only the position of the repetition in the three-syllabic sequence is varied. Humans are particularly sensitive to rules that are expressed as a repetition of an element at the edges of a sequence (Endress et al., 2005). In our experiment, repetitions were always at the edge of the sequence. It is thus unclear as to what extent the present results in rats can be generalized to other types of rules. Furthermore, the types of rules applied to the previous studies on rule extraction have been under debate (Gentner et al., 2010; ten Cate et al., 2010). Thus, far studies in song birds have been progressive in solving this problem (e.g., van Heijningen et al., 2013), but there are still open questions (ten Cate and Okanoya, 2012). Electrophysiological methods which provide accurate information on the timing of neural activity (recorded in animals and humans) would be a feasible addition when studying different levels of cognitive complexity required in rule extraction. In humans, event-related potentials to study processing of non-adjacent dependencies, i.e., AXC structure in which the first and the last element are dependent (De Diego Balaguer et al., 2007; Mueller et al., 2009) and structural rules (ABB vs. ABA, Sun et al., 2012) in speech sounds have been utilized.

Previous behavioral research has failed to find evidence for rule extraction from speech sounds in rats (Toro and Trobalon, 2005). In this study, rats were presented with similar three-syllabic sequences of speech sounds, as in Marcus et al. (the third experiment, 1999). Our stimuli were nearly identical and the variability in the “standard” and “deviant” sequences was also the same (16 standard variants and 2 deviant variants of both deviant types). In the study by Toro and Trobalon (2005), rats indicated the detection of the pattern violation by pressing a lever. The present positive finding may be related to the methodology used. Namely, the mismatch response is known to be capable of probing into auditory cognition regardless of its behavioral manifestations (Tremblay et al., 1998). This method can bypass a wide range of factors related to behavior, for example, motivation, attention, or requirements of overt behavior. However, the constraints of such non-behavioral measures should also be acknowledged. Namely, it is unclear whether this ability can support behavioral adaptation in rats or not. Nevertheless, its existence in an animal species, which do not use complex sequences of calls in intra-species communication, (as compared to human speech or birdsong, e.g., Doupe and Kuhl, 1999; Gentner et al., 2006) supports the notion of its non-linguistic origin. Moreover, these findings endorse the view that even the most complex functions, quintessentially considered inherent to the human brain only, may in fact, also be represented in a primitive form (Näätänen et al., 2010) in brains thus far considered evolutionarily incapable of such procedures. Since extraction of rule-like patterns, in serially presented spectro-temporally complex sounds, is one of the mechanisms utilized by humans in receptive language learning the results might imply that some of the mechanisms supporting human language learning may not have evolved solely for human language during evolution.

In conclusion, the present results demonstrate the ability of the anesthetized rat brain to detect and represent the common abstract rule or pattern obeyed by a sequence of speech-like sound stimuli with a wide acoustic variation. Hence, these results appear to give a major contribution to the evidence suggesting the presence of the automatic sensory-cognitive core of cognitive function that is shared by humans and different other, at least higher species, at different developmental stages, and even in different states of consciousness, as proposed by Näätänen et al. (2001, 2010).

## Author contributions

All authors contributed substantially to the conception and design of the work; Tanel Mällo, Timo Ruusuvirta, and Piia Astikainen contributed to the acquisition and analysis of the data, and all authors contributed to the interpretation of data for the work; Tanel Mällo and Piia Astikainen drafted the work, and Timo Ruusuvirta and Risto Näätänen contributed to revising it critically for its important intellectual content. Final approval of the version to be published was attained from all authors who also agreed to be accountable for all aspects of the work in ensuring that questions related to the accuracy or integrity of any part of the work are appropriately investigated and resolved.

### Conflict of interest statement

The authors declare that the research was conducted in the absence of any commercial or financial relationships that could be construed as a potential conflict of interest.
